# Pennsylvania Hospital

**Published:** 1842-01-29

**Authors:** 


### CLINICAL REPORTS.

### PENNSYLVANIA HOSPITAL—*Surgical Wards—Service of* DR. E. PEACE.

*Fractured Thigh.*—J. S., ætat. 11, was admitted Nov. 10th, with an oblique fracture of the middle third of the right thigh-bone, produced by a blow upon the part. When first seen, about an hour and a half after the accident, the limb was shortened at least two inches. It was dressed, as usual, with Physick's modification of Desault's apparatus. The boy's skin was so delicate and sensitive as in a few hours to require the relaxation of the extending and counter-extending bands, although they had been applied with great care. On the third or fourth day, in consequence of the right groin having suffered some abrasion from the perineal band of Physick's splints, Hartshorne's splints were substituted, and the counter-extension effected by means of the cushioned inner splint of the latter apparatus upon the perineum of the left side. It was found to be impossible, however, to maintain the extension even in this manner for any length of time, on account of the pain caused by the pressure upon the heel, ankles, and instep of the gaiter to which the extending bands were attached.

These latter were loosened, therefore, and the gaiter used only to preserve the upright position of the foot. The thigh was then subjected to firm and uniform compression by means of a pillow and short splints within the long ones already employed, in addition to a roller applied in the ordinary manner to the whole extremity. Union, which appeared to be well advanced on the twentieth day, had become quite firm at the end of twenty-five days. The splints were removed at the expiration of the fourth week, the roller being continued. After resting one week longer, the patient was allowed to move about on crutches, without using the limb. The crutches were gradually laid aside in the course of the succeeding two weeks. The boy was discharged January 12th, cured, without perceptible deformity or lameness, not more than a quarter of an inch in shortening having been detected by the most careful and repeated measuring.

2. *Concussion of the Brain and Fractured Clavicle.*—M. K., ætat. 19, admitted with severe concussion of the brain and oblique fracture of the middle third of the right clavicle, the effects of a fall of about six feet upon a stone pavement. When brought in, the patient appeared to be in a deep and quiet sleep, from which it was impossible entirely to rouse him.

Considerable prostration was also observed. Reaction having been fully established under the influence of artificial heat and sinapisms to the extremities, blood was freely taken from the head by means of cups, and a purgative enema was administered. The symptoms of concussion continued gradually disappearing for five days; during which time cathartics and diaphoretics were given, the cups were re-applied, and a rigidly low diet and perfect rest enjoined. No inflammation of the brain supervened. Owing to the cerebral complication, it was very difficult properly to reduce and adjust the fragments of the broken clavicle—especially as they were very much displaced—until the third day; when the fracture was securely dressed with Fox's clavicle apparatus. Consolidation was complete, without visible deformity, on the twenty-first day. The patient was discharged January 14th, cured. This is the fifth case of fractured clavicle treated in these wards during the last five months.

The four cases preceding this were all treated, according to the custom of the house, with Fox's apparatus. In the first of them, the patient being twenty-four years of age, union took place in about twenty days, a slight prominence remaining at the seat of fracture. In the second, the case of an individual over fifty years, the bones did not unite before the twenty-eighth or thirtieth day, but there was no deformity whatever. The two other cases left the hospital while under treatment, and therefore afforded no results.

The case of M. K. is also the second instance of simple fracture complicated with concussion of the brain treated in these wards during the last five months.

The first, although in a subject only eleven years old, was of a far more serious nature than the present, inasmuch as the concussion of the brain was severe, and was succeeded by a violent and nearly fatal inflammation of that organ, while there were two fractures, one of the lower jaw, and one of the left humerus. Although the violent delirium and constant restlessness of the boy seriously interfered with the treatment of the fractures, putting utterly out of the question, in fact, any thing like rest of the fragments, and especially of those of the lower jaw, upon which no dressing could be long retained. Yet the latter became firm in sixteen days, without deformity, and the humerus united within thirty days also without displacement. In this case, besides the constant delirium, marked by extreme restlessness and a disposition to tear off every bandage, there was, during several days, alarming prostration of the vital powers. It is a matter of regret to the reporter that this case was treated during his temporary absence from the house, and that he is, therefore, unable to furnish more than the above meagre details.

